# DNA methylation at quantitative trait loci (mQTLs) varies with cell type and nonheritable factors and may improve breast cancer risk assessment

**DOI:** 10.1038/s41698-023-00452-2

**Published:** 2023-09-27

**Authors:** Chiara Herzog, Allison Jones, Iona Evans, Michal Zikan, David Cibula, Nadia Harbeck, Nicoletta Colombo, Angelique Flöter Rådestad, Kristina Gemzell-Danielsson, Nora Pashayan, Martin Widschwendter

**Affiliations:** 1 European Translational Oncology Prevention and Screening (EUTOPS) Institute, Milser Str. 10, 6060 Hall in Tirol, Austria; 2 Research Institute for Biomedical Aging Research, Universität Innsbruck, 6020 Innsbruck, Austria; 3 Department of Women’s Cancer, UCL EGA Institute for Women’s Health, University College London, Medical School Building, Room 340, 74 Huntley Street, WC1E 6AU London, UK; 4 Department of Gynecology and Obstetrics, Charles University in Prague, First Faculty of Medicine and Hospital Na Bulovce, Prague, Czech Republic; 5 Gynaecologic Oncology Center, Department of Obstetrics and Gynecology, First Faculty of Medicine, Charles University in Prague, General University Hospital in Prague, Prague, Czech Republic; 6 Breast Center, Department of Obstetrics and Gynecology and CCC Munich, LMU University Hospital, Munich, Germany; 7 Istituto Europeo di Oncologia, Milan, Italy; 8 University of Milano-Bicocca, Milan, Italy; 9 Department of Women’s and Children’s Health, Karolinska Institutet, Stockholm, Sweden; 10 Department of Applied Health Research, University College London, London, UK

**Keywords:** Breast cancer, Epigenetics

## Abstract

To individualise breast cancer (BC) prevention, markers to follow a person’s changing environment and health extending beyond static genetic risk scores are required. Here, we analysed cervical and breast DNA methylation (*n* = 1848) and single nucleotide polymorphisms (*n* = 1442) and demonstrate that a linear combination of methylation levels at 104 BC-associated methylation quantitative trait loci (mQTL) CpGs, termed the WID™-qtBC index, can identify women with breast cancer in hormone-sensitive tissues (AUC = 0.71 [95% CI: 0.65–0.77] in cervical samples). Women in the highest combined risk group (high polygenic risk score and WID™-qtBC) had a 9.6-fold increased risk for BC [95% CI: 4.7–21] compared to the low-risk group and tended to present at more advanced stages. Importantly, the WID™-qtBC is influenced by non-genetic BC risk factors, including age and body mass index, and can be modified by a preventive pharmacological intervention, indicating an interaction between genome and environment recorded at the level of the epigenome. Our findings indicate that methylation levels at mQTLs in relevant surrogate tissues could enable integration of heritable and non-heritable factors for improved disease risk stratification.

## Introduction

Reliable tools to risk-stratify women for primary and secondary breast cancer preventive measures are urgently needed^1,2^. Since 2007^3^, genome-wide association studies (GWAS) have identified hundreds of single nucleotide polymorphisms (SNPs) associated with breast cancer risk. A combination of 313 SNPs (polygenic risk score, PRS_313_) has previously been reported to achieve an area under the receiver-operator curve (AUC) of 0.630 (95%CI: 0.628–0.651) overall, and 0.641 and 0.601 for estrogen receptor (ER) positive (ER+) and negative (ER-) cancers, respectively^4^. This PRS_313_ improves discrimination over age and clinical risk factors in initial analyses^5^, and is being implemented in primary and secondary breast cancer preventive strategies^1,2,6,7^. While PRSs have been suggested to offer promise in improving clinical risk stratification, they suffer from several shortcomings. First, PRSs often show varying performance in non-European-ancestry populations^8,9^ due to being derived from GWAS in populations of largely European descent, although limited portability can be overcome by including more diverse populations and improved technology, deriving arrays to cover diverse variants, and efforts are underway to recalibrate PRSs for all ancestries^10^. Second, a major limitation of PRSs is that they only reflect static genetic risk and do not account for environmental, socioeconomic, or lifestyle risk factors, or their interplay with genetic risk factors, which constitute important modifiable factors and targets for disease prevention^11^. For instance, childhood postcode as an indicator of socioeconomic inequalities has been found to perform equally well as a predictor of risk for most common diseases as most polygenic risk scores^12^. The PRS can subsequently also not be used to monitor changes in risk over time^11^. Even the most predictive PRSs have been suggested to explain only a low proportion of the overall variance for a trait in each population^13^, and a recent study raised concern about the potential applicability of PRSs^11,14^. Biomarkers and risk scores capable of capturing and integrating both genetic and modifiable risk factors, for instance DNA methylation (DNAme), may therefore be suitable to extend risk prediction beyond the limitations of PRSs.

DNAme changes have been observed in histologically normal breast tissue adjacent to breast cancers^15^. This ‘epigenetic field defect’ may be elicited by both genetic and non-genetic factors including lifestyle, reproductive, and environmental exposures contributing to breast cancer development^16^, and could be exploited for risk stratification, yet breast samples require invasive biopsies and are therefore not feasible for routine clinical screening. Several proof of principle studies, almost exclusively performed in blood, have demonstrated that certain DNAme changes in a non-invasively collected ‘surrogate’ tissue could also be associated with breast cancer predisposition^17–22^. Steroid hormones play an essential role in breast cancer formation, as prolonged^23^ or higher than average^24^ exposure to progesterone are strongly associated with the formation of poor prognostic breast cancer^23–28^, and the progesterone receptor antagonist mifepristone effectively prevents breast cancer formation in mice^29^. To record steroid hormone exposure and possible other factors contributing to breast cancer formation, the surrogate sample in which DNAme is measured for risk detection should ideally resemble the cell of origin for the cancer, i.e., a hormone-sensitive epithelial cell. It has recently been shown that trait-linked methylation quantitative trait loci (mQTLs) exhibit molecular regulatory pleiotropy and enrichment in trait-relevant tissues^30^, and in previous work we demonstrated that DNAme patterns in cervical samples can indicate current or future women’s cancers, including anatomically distant breast and ovarian cancers^31,32^. Cervical samples are possibly suitable surrogate samples for women’s cancer detection as they contain hormone-sensitive epithelial cells similar to the tissue at risk (breast, ovary), and can be obtained without a tissue biopsy, but it has not been systematically assessed whether cervical samples are better suited for breast/ovarian cancer risk prediction than other promising non-invasive surrogate samples such as buccal samples or blood.

Here, we compare the suitability of three surrogate sample types - cervical, buccal, and blood - to reflect breast DNAme variability, develop a new mQTL-based predictor for breast cancer risk, and investigate the interplay of genetic and non-genetic factors using CpG sites whose DNAme levels are strongly associated with breast cancer SNPs (mQTLs)^33^.

## Results

### Identification of the most informative surrogate sample to reflect breast DNAme variability

**Fig. 1:**
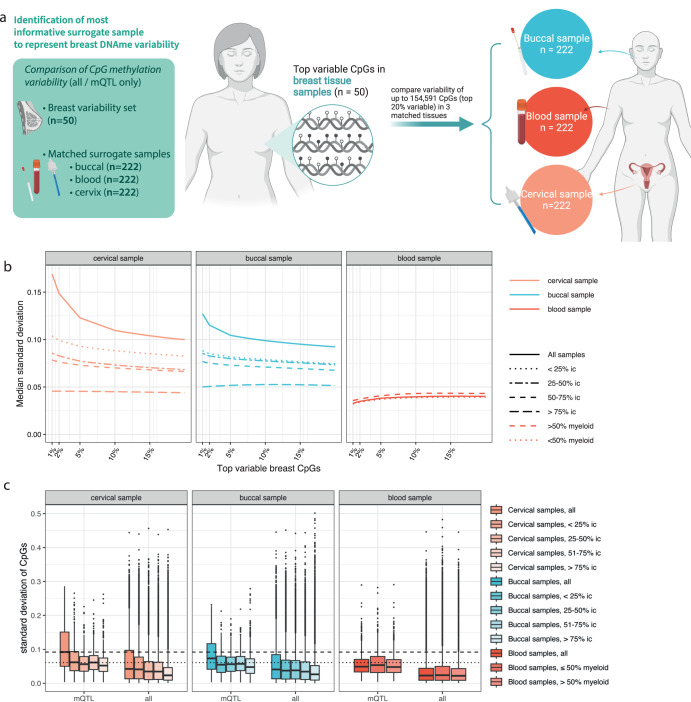
Identification of the most informative surrogate sample for breast-variable DNA methylation indicates that cervical samples exhibit higher variability in the top-variable breast CpGs compared to matched buccal and blood samples. **a** Higher variability indicates the potential presence of more information, opposed to CpGs which are homogeneously methylated or unmethylated across samples. We explored this by identifying variability of CpGs in tissue at risk of breast cancer ( = breast tissue) and assessing variability of these top breast-variable CpGs in three non-invasive surrogate tissues, utilising matched buccal, blood, and cervical samples from the same individuals (*n* = 222 per tissue). **b** Standard deviation of the top variable breast CpGs (1, 2, 5, 10, 15, and 20 percentiles, respectively) in matched cervical, buccal, and blood samples (all and separated by inferred immune cell composition). **c** Variability of all CpGs versus mQTL CpGs in the three matched tissues (all or separated by inferred immune cell composition). The dashed line shows median variability of all CpGs in all cervical samples while the dotted line shows median variability of mQTLs in cervical samples with an immune cell composition (ic) < 25%. Boxplot boxes indicate median (centre line), interquartile range (bounds of box), and 95% confidence interval (whiskers). DNAme DNA methylation, mQTL methylation quantitative trait locus, SNP single nucleotide polymorphism, ic immune cell (proportion).

We initially analysed DNAme variability at 772,955 CpG sites in 50 normal breast tissue samples and, using a matched set of three less-invasive potential ‘surrogate’ samples – cervical, buccal, and blood – from 222 healthy volunteers (*n* = 113 *BRCA1/2* wild type and *n* = 109 *BRCA1/2* mutant), assessed which surrogate tissue best represents the breast tissue DNAme variability, accounting for cell type heterogeneity (see Methods section) (Fig. 1a). The top breast-variable CpGs showed higher variability in epithelial fractions of cervical and buccal samples compared to immune fractions or blood samples (Fig. 1b), indicating samples containing hormone-sensitive epithelial cells might be a better suitable proxy for breast tissue than immune-based samples. mQTLs are potentially best identified in tissues with low variability and low susceptibility to non-genetic risk factors like hormones (i.e., blood) and then assessed in surrogate tissues whose variability is closest to the tissue at risk and which can capture the impact of non-genetic factors at these mQTLs. Utilising peripheral blood, Ho et al.^33^ identified 822 mQTLs for 235 of the 313 breast cancer PRS variants with minor allele frequencies > 5%. Amongst 822 mQTL CpGs, 704 were available in our datasets after quality control. We assessed whether the variability of these 704 mQTL CpGs followed the same pattern as the breast-variable CpGs. Indeed, these mQTL CpGs were generally more variable than average variability across all tissues but showed the highest variability in cervical samples (Fig. 1c). Variability across cervical and buccal samples decreased with increasing immune cell content, again highlighting higher variability in epithelial than immune samples (Fig. 1c).

### Utilisation of DNAme in cervical samples to develop an mQTL-based breast cancer risk predictor

**Fig. 2:**
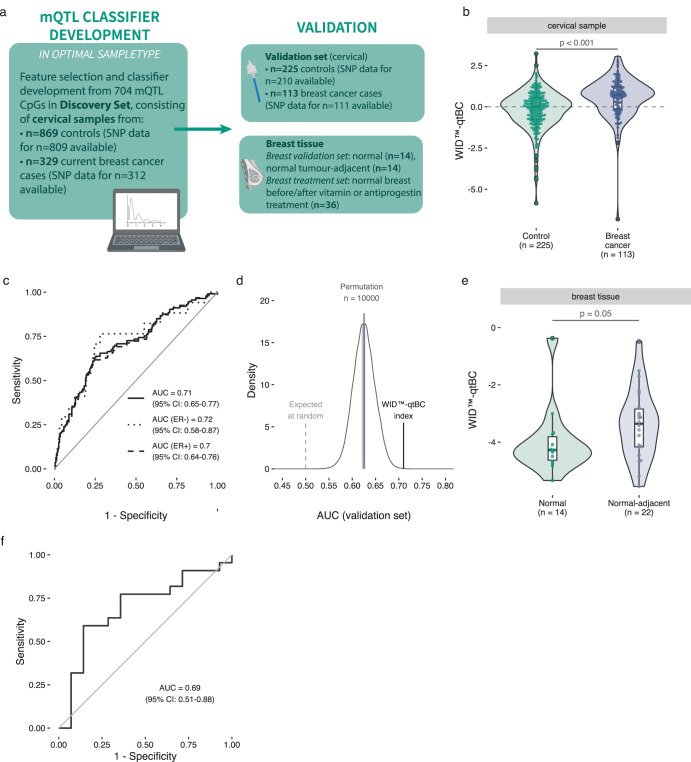
The WID™-qtBC index distinguishes breast cancer cases and controls in cervical and breast samples. **a** Outline of classifier development and validation strategy. **b** The WID™-qtBC index is increased in cervical samples from current breast cancer cases compared to controls in the validation set (*p* = 1.2e-07 in two-tailed Student’s *t*-test). **c** The WID™-qtBC has an AUC of 0.71 and is unaffected by estrogen receptor (ER) status. **d** Permutation analysis of index training featuring randomly selected CpGs indicates that the WID™-qtBC AUC in the validation set is higher than expected by chance. Shaded area indicates 95% confidence interval from permutation testing. **e** The WID™-qtBC index is increased in normal tissue adjacent to breast cancer compared to normal breast tissue. **f** ROC curve for discrimination of normal to normal-adjacent tissue based on the WID™-qtBC index. Boxplot boxes indicate median (centre line), interquartile range (bounds of box), and 95% confidence interval (whiskers). AUC area under the receiver operating characteristic curve, ER- estrogen receptor negative breast cancer, ER+ estrogen receptor positive breast cancer.

We evaluated whether a cervical sample mQTL-based predictor could be used to identify breast cancer cases (Fig. 2a). To enrich for clinically significant breast cancer cases, we only included samples from cases (*n* = 442, for *n* = 423 SNP data were available) that had at least one poor prognostic feature (tumour >2 cm, node positive, grade 3, or ER negative; detailed characteristics shown in Supplementary Table 2). We used ridge or lasso regression to develop a breast cancer classifier based on 704 mQTLs or an informative subset thereof, respectively, in a training dataset of 572 cancer-free controls and 217 breast cancer cases. This training set was based on two thirds of samples in the discovery set (see Supplementary Fig. 1, Supplementary Table 1). The remaining samples in the discovery set were used as an internal validation set (297 controls, 112 cases) to determine the optimal number of CpGs used to construct the index. Lasso regression, resulting in 104 non-zero CpG coefficients, achieved in slightly higher discriminatory performance than ridge regression which used all 704 CpGs (AUC: 0.68, 95% CI: 0.62–0.73, versus 0.66, 95% CI: 0.60–0.72, respectively). The parameter-optimised final version of the index, the Women’s cancer risk IDentification – quantitative trait Breast Cancer (WID™-qtBC) index is a linear combination of the 104 CpGs based on lasso regression that was finalised by training on the entire discovery set (*n* = 869 cancer-free controls, *n* = 329 cancer cases) and validated in an independent validation dataset consisting of cervical samples from 225 controls and 113 breast cancer cases (Supplementary Fig. 1). The full list of CpGs including coefficients is presented in Supplementary Table 3.

In the validation dataset, we observed a significantly higher WID™-qtBC in women with breast cancer compared to controls (Fig. 2b, *p* = 1.2e-07, two-tailed Student’s t-test). The WID™-qtBC obtained an AUC of 0.71 (Fig. 2c; 95% CI: 0.65–0.77), and was not strongly dependent on ER status (ER-negative [*n* = 17]: 0.72 [95% CI: 0.58–0.87], ER-positive [*n* = 94]: 0.70 [95% CI: 0.64–0.76]) (Fig. 2c). To assess the specificity of the index we performed 10,000 permutations to randomly select 704 non-mQTL CpGs out of all IlluminaMethylationEPIC CpGs and allowing lasso regression to select informative CpGs for each permutation. The median AUC across the 10,000 permutations was 0.62 (Fig. 2d), demonstrating that although other non-mQTL CpGs retain some information indicative of case and control status ( ~ 14% of all CpGs on the array are significantly associated with BC status before adjustment for multiplicity^31^), the WID™-qtBC AUC in the external validation set is higher than expected by chance.

### Cervical mQTL predictor WID™-qtBC distinguishes normal breast tissue from breast tissue adjacent to a cancer

We were next interested whether the cervical classifier would be able to distinguish normal breast tissue from breast tissue adjacent to a cancer. The WID™-qtBC was elevated in normal tissue surrounding breast cancer compared to normal tissue from unaffected women (Fig. 2e) and could discriminate normal tissue adjacent to cancer from normal breast tissue with an AUC of 0.69 (95% CI:0.49–0.87) (Fig. 2f).

### Investigating the interaction between WID™-qtBC and PRS_313_ for enhanced risk stratification

**Fig. 3:**
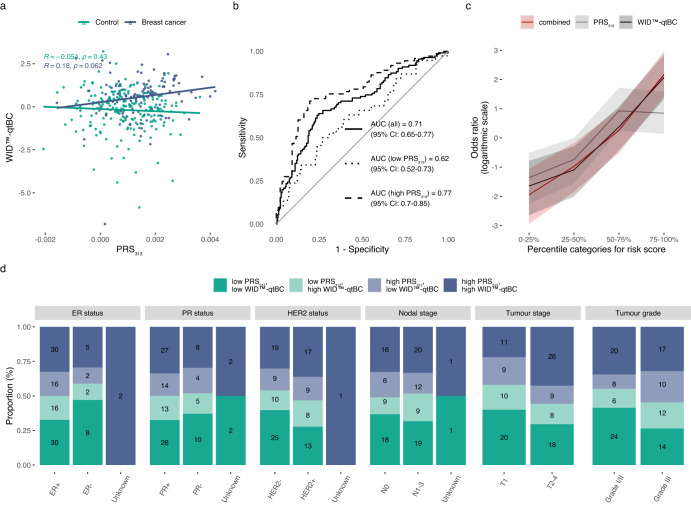
Association of the WID^TM^-qtBC with the 313 SNP polygenic risk score and cancer characteristics. **a** Pearson correlation of the WID^TM^-qtBC index with the polygenic risk score (PRS_313_) in the Validation set. **b** ROC curve analysis of the WID^TM^-qtBC index stratified by median PRS group (higher or lower than median) in the Validation set. **c** Odds ratio of the PRS_313_, WID^TM^-qtBC, or their combination, across different percentile categories for the risk scores. Shading indicates 95% confidence intervals. **d** Assessment of estrogen receptor (ER), progesterone receptor (PR), and human epidermal growth factor receptor 2 (HER2 status), nodal and tumour stage, and tumour grade amongst cases in the Validation set, comparing different risk groups defined in Table 1. Numbers in bars indicate n for each group. *p* = 0.0349 for an association of T2-4 tumours with increasing PRS_313_ and WID™-qtBC, *p* = 0.0167 for increasing Grade III tumours with increasing PRS_313_ and WID™-qtBC. *P* values were derived from logistic regression model using ER status, PR status, HER2 status, nodal stage, tumour stage, and tumour grade as independent variables and risk group as the dependent variable. ER status was not significant. Abbreviations: PRS_313_ polygenic risk score (313 SNPs). AUC area under the receiver operating characteristic, ER estrogen receptor, PR progesterone receptor.

We evaluated the association between genetic predisposition, based on the PRS_313_, and the mQTL-based classifier. Surprisingly, although mQTLs were identified based on genetic variants in the PRS_313_, the WID™-qtBC exhibited limited correlation with PRS_313_ (Fig. 3a). Nonetheless, the WID™-qtBC performed better in individuals with a higher PRS_313_ score compared to a lower PRS_313_ score (stratifying by median PRS_313_) (Fig. 3b), indicating a potential interaction between the two.

When comparing risk stratification efficacy based on median values of either the PRS_313_ or WID™-qtBC, the higher risk groups exhibited odds ratios of 2.7 (95% CI: 1.7–4.4) (PRS_313_, Supplementary Table 4) or 3.9 (95% CI: 2.4–6.5) (WID™-qtBC, Supplementary Table 5) compared to the lower risk groups, indicating that the WID™-qtBC may offer a small benefit in risk stratification compared to the PRS_313_, although this was not significant.

We next assessed whether combination of the WID™-qtBC and the PRS_313_ might enhance risk stratification (Table 1). Individuals with both a high PRS_313_ and high WID™-qtBC index, as defined by values higher than the median, had a significantly increased odds ratio for being diagnosed with breast cancer in both the discovery and validation sets (Table 1; discovery set OR (high PRS, high WID™-qtBC) = 26, 95% CI: 15–47; validation set OR (high PRS, high WID™-qtBC) = 9.6, 95% CI: 4.7–21). The higher OR in the discovery set is likely a reflection of the fact that the WID™-qtBC was trained in this set and is thus not representative, while values in the validation set may be more representative.

**Table 1:** Significantly increased risk of breast cancer for individuals with both a high PRS_313_ and high WID™-qtBC index.

Risk group	Cases (*n*)	Controls (*n*)	Odds ratio	95% CI	*P* value
**Discovery set**					
low PRS_313_, low WID™-qtBC	16	301	1 (Reference)	–	–
high PRS_313_, low WID™-qtBC	23	221	**1.9**	**1**–**3.9**	***p*** = **4.61e-02**
low PRS_313_, high WID™-qtBC	89	155	**11**	**6.2**–**19**	***p*** = **7.26e-22**
high PRS_313,_ high WID™-qtBC	184	132	**26**	**15**–**47**	***p*** = **3.04e-52**
**Validation set**					
low PRS_313_, low WID™-qtBC	14	70	1 (Reference)	–	–
high PRS_313_, low WID™-qtBC	18	59	1.5	0.69–3.4	*p* = 3.26e-01
low PRS_313_, high WID™-qtBC	24	53	**2.2**	**1.1**–**4.9**	***p*** = **4.06e-02**
high PRS_313,_ high WID™-qtBC	55	28	**9.6**	**4.7**–**21**	***p*** = **4.80e-11**

Cut-offs were defined based on the median PRS_313_ and WID™-qtBC (high scores were above the median). *P* values and odds ratios were estimated using median-unbiased estimation. Statistically significant values are shown in bold.

Low and high grouping was defined on median values of the respective risk score in each set. High scores were above the median, while low scores were defined as equal to or below the median.

Comparing the PRS_313,_ WID™-qtBC, or a combination thereof across different risk quartiles, the WID™-qtBC either alone or in combination with the PRS_313_ exhibited an increased OR compared to the PRS_313_ in the highest risk quartile, suggesting enhanced risk stratification (Fig. 3c); we did not see an improved performance when combining the WID™-qtBC with the PRS_313_ compared to the WID™-qtBC alone in this set, although sample numbers were small (Fig. 3c).

### Association of combined WID™-qtBC and PRS_313_ risk groups with clinical features

The highest risk scores (both high PRS_313_ and high WID™-qtBC) were associated with higher tumour stages and grades (Fig. 3d, *p* = 0.0349 and *p* = 0.0167 for stage and grade, respectively, derived from logistic regression model using ER status, PR status, HER2 status, nodal stage, tumour stage, and tumour grade as independent variables and risk group as the dependent variable). Of note, the proportion of patients with a low PRS and a low WID™-qtBC appeared higher in the ER- than in the ER+ group, which would not clinically be expected. In a logistic regression model, either accounting or not accounting for additional receptor status and stage/grade, ER status was associated with an increase in risk group, but this was not significant. We therefore believe this to reflect the relatively small sample size rather than a clinically significant effect. Due to the case/control design our study population is not entirely reflective of the general population, and a prospective collection of cohort samples will be required to further evaluate the use of the WID™-qtBC.

### Malleability of WID™-qtBC by external factors

**Fig. 4:**
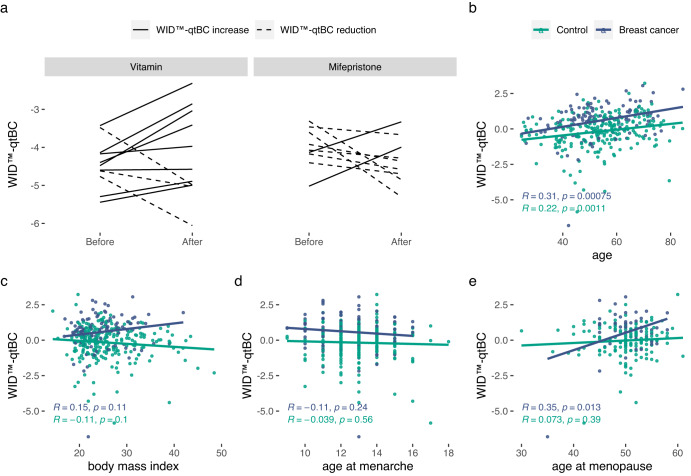
Dynamic changes of the WID^TM^-qtBC and association with non-genetic characteristics. **a** Matched breast biopsy samples in healthy women before and after two months of vitamin or mifepristone treatment. 7/9 (77.8%) women in the mifepristone group showed a reduction in the WID^TM^-qtBC while only 3/11 (27.3%) showed a reduction in the vitamin group. Association of the WID™-qtBC scores in cervical samples in the validation set with (**b**) age, (**c**) body mass index, (**d**) age at menarche, and (**e**) age at menopause in cancer cases and controls (R indicates Pearson correlation coefficient).

Whereas the PRS_313_ captures the genetic heritable risk, the WID™-qtBC could be modified by non-heritable factors as it is based on DNA methylation, which is known to be influenced by ageing and external exposures^34^. This might make it amenable to dynamic risk monitoring. Inhibition of the action of progesterone, a key risk driver of breast cancer development, has been suggested to reduce breast cancer risk^35^. Two-month exposure to mifepristone reduced the WID™-qtBC in breast tissues of 7/9 individuals compared to a reduction in only 3/11 women in a control group treated with vitamins (Fig. 4a), although this was not significant using paired Wilcoxon tests (*p* = 0.31 and *p* = 0.43 for mifepristone and vitamin use, respectively). Interestingly, the WID™-qtBC also was significantly positively associated with age, body mass index, and age at menopause (the latter two only in breast cancer cases) (Fig. 4b-e) in cervical samples of the validation set, indicating an interaction between genetic (mQTLs) with non-genetic risk factors. A high PRS_313_ appeared to drive higher WID™-qtBC levels particularly in younger women that were aged below the median age in the validation set (*p* = 0.017 compared with lowest PRS_313_ quartile in Wilcoxon test, Supplementary Figure 2).

## Discussion

Our study demonstrates that a combination of 104 mQTL CpGs can identify women with breast cancer using hormone-sensitive surrogate samples (cervical sample; Fig. 2b, c) or breast tissue (Fig. 2e, f). A recent clinical trial showed that breast magnetic resonance imaging (MRI) detected cancers at earlier stages (smaller and less likely node-positive) in women aged 30–55 years with a cumulative lifetime breast cancer risk of at least 20% than mammography^36^, and that MRI screening is preferred in high-risk women^37^. Hence, taking the distribution of controls in the validation set as a proxy for the general population, offering the 13.8% of women within the high PRS_313_/high WID™-qtBC group screening will result in identification of 50.4% of all breast cancers, and these will likely be cancers which might benefit most from earlier detection, e.g., using MRI or Digital Breast Tomosynthesis.

Our results indicate that mQTL sites may be sensitive to environmental impacts and cancer driving mechanisms in a cell-type specific manner (Figs. 1b and 4), and could thereby act as a proxy for the cumulative exposure to steroid sex hormones and other risk factors that are essential for the development of both ER+ (evidenced by the primary preventive effects of tamoxifen and aromatase inhibitors^38,39^) and ER- breast cancers (evidenced by the numerous observational and experimental studies^40^).

Additional studies will be required to assess whether convenience and acceptability of obtaining a WID™-qtBC index can be further increased by utilising a self-collected cervicovaginal sample, a strategy well-accepted for cervical screening^41^. A side-by-side comparison of WID™-qtBC with the PRS_313_ and study of their benefits – individually or in a combined score – across different risk grouping strategies in prospectively collected samples from diverse ethnic populations will be required to motivate the use of the WID™-qtBC in a clinical setting. Moreover, samples predating diagnosis will be required to assess how long in advance of breast cancer onset the WID™-qtBC index could identify at-risk women and whether it would be suitable for implementation into primary preventive strategies – both as a risk indicator and a tool to monitor the efficacy of primary preventive measures due to its dynamic nature. Further studies will also need to evaluate the real-world sensitivity and specificity to avoid overdiagnosis, a common shortfall of PRS screening that can cause negative emotions for patients and substantial financial burden for healthcare providers^11^.

The significance of our findings should also encourage other groups to assess mQTLs for other diseases in surrogate tissues which are histologically and biologically more closely connected to the organ at risk.

## Methods

### Study overview

An overview of samples used in this study is shown in Supplementary Table 1. We initially identified the top variable CpGs in *N* = 50 breast samples (“breast variability set”) from healthy volunteers (*n* = 14), BRCA1/2 mutation carriers undergoing risk-reducing surgery (*n* = 14) or normal tissue adjacent to triple negative cancers (*n* = 22), and evaluated them in *n* = 666 samples from 222 women for which matched cervical, buccal, and blood samples were collected (“matched surrogate samples”). To derive a new classifier index based on mQTL loci, samples from the FORECEE case-control study^31^ were utilised: “discovery set” samples were collected with the intent of index development, while the “validation set” was only used for index validation. The WID™-qtBC was evaluated in *n* = 40 normal breast tissue samples before and after vitamin or mifepristone treatment (“breast treatment set”), as well as normal breast samples (*n* = 14) and normal breast samples adjacent to triple negative cancers (*n* = 22) (subset of the “breast variability set”). Of note, the “breast variability set” did not influence the risk score development or training, it was solely used to highlight differential variability of CpGs in different tissues.

### Breast tissue samples

#### Breast variability set (n = 50)

Breast variability set samples were derived from two sources. The first set contained a total of 42 breast samples from premenopausal women aged 19–54 years: normal breast tissue from 14 women who underwent cosmetic breast operations, normal breast tissue from women who underwent prophylactic mastectomies due to a *BRCA1* (*n* = 10) or a *BRCA2* (*n* = 4) mutation, and 14 normal samples from women who underwent surgery for triple-negative breast cancer (normal tissue adjacent to the cancer was collected). All samples were collected fresh from theatre and processed within 1 h of surgical excision. Fresh samples were rapidly frozen in Liquid Nitrogen and stored at −80° C. Ethical approval was obtained from the NRES Committee East of England (reference number 15/EE/0192). The second set contained an additional *n* = 8 normal-adjacent samples (‘ipsilateral-normal’) from Gene Expression Omnibus dataset GSE133985.

#### Breast validation set (n = 36)

The breast validation set was a subset of the breast variability set and comprised normal (*n* = 14) and normal tissue adjacent to a triple-negative breast cancer (*n* = 14) from the breast variability set plus an additional *n* = 8 normal-adjacent samples (‘ipsilateral-normal’) from Gene Expression Omnibus dataset GSE133985.

#### Breast treatment set (n = 40)

Normal breast tissue samples before and after two months of Vitamin (*n* = 22 samples at two timepoints) or anti-progestine Mifepristone (50 mg every other day, *n* = 18 samples from two timepoints)) from the trial “*Mifepristone treatment prior to insertion of a levonorgestrel releasing intrauterine system for improved bleeding control – a randomised controlled trial*” (EudraCT number: 2009-009014-40) were obtained as previously described in ref. ^35^.

### Matched surrogate samples

Matched cervical, buccal, and blood samples from 222 healthy volunteers were part of the FORECEE programme (Female cancer prediction using cervical omics to individualise screening and prevention - 4 C)^42^, a multi-centre study involving several recruitment sites in five European countries (the UK, Czech Republic, Italy, Norway, and Germany). The FORECEE programme had ethical approval from the UK Health Research Authority (REC 14/LO/1633) and all other contributing centres^42^. Participants were aged >18 years and <86 years. After signing an informed consent, participants completed a pre- and post-enrolment questionnaire.

Cervical liquid-based cytology samples were collected at appropriate clinical venues by trained staff using the ThinPrep system (Hologic Inc., cat #70098-002). Cervical cells were sampled from the cervix using a cervix brush (Rovers Medical Devices, cat #70671-001), which was rotated 5 times through 360 degrees whilst in contact with the cervix to maximise cell sampling. The brush was removed from the vagina and immersed in a ThinPrep vial containing Preserve-cyt fluid and then pushed against the bottom of the vial 10 times to facilitate release of the cells from the brush into the solution. The sample vial was sealed and stored locally at room temperature. Buccal cells were collected using two Copan 4N6FLOQ Buccal Swabs (Copan Medical Diagnostics, cat #4504 C) by firmly brushing the swab head 5-6 times against the buccal mucosa of each cheek. The swabs were re-capped and left to dry out at room temperature within the sampling tube which contains a drying desiccant. 2.5 ml of venous whole blood was collected in PAX gene blood DNA tubes (BD Biosciences #761165) and stored locally at 4 °C. All samples were shipped to University College London (UCL) at ambient temperature. Biological samples were given an anonymous Participant ID Number, which was assigned to the person’s name in a securely stored link file.

### Discovery and validation sets

Samples from the case-control study (442 breast cancer cases and 1094 matched cancer-free controls, Supplementary Figure 1) were part of the FORECEE programme (described above), and all abovementioned criteria applied. Women diagnosed with breast cancer (case) or a non-malignant benign gynaecological condition (control) were recruited from outpatient hospital clinics in the five study sites, while the healthy volunteers were recruited through outreach public engagement campaigns in the UK. Women with a current diagnosis of (a) primary breast cancer with poor prognosis features (Grade III and/or T2/3 and/or N1/2 and/or hormone receptor positive) were recruited prior to receiving any systemic treatment (chemo- or endocrine or trastuzumab, etc.) or surgery or radiotherapy. Details of cancer histologies are outlined in Supplementary Table 2.

The discovery set controls were initially matched one-to-one with cases based on menopausal status, age (5-year age ranges where possible), and recruitment centre/country. However, due to an imbalance in recruitment of cases and controls at some centres, a number of cases were matched on age and menopausal status alone. Cancer histological data was collected post-recruitment either by clinicians directly involved in the diagnosis/treatment of the cancer cases or by a nominated data manager with access to the in-house hospital systems.

### Sample processing and DNA extraction

When preparing for sample storage in the laboratory, cervical samples were poured into 50 ml Falcon tubes and left to sediment at room temperature for 2 h. 1 mL wide bore tips were then used to transfer the enriched cellular sediment into a 2 mL vial. The cervical sediments were washed twice with PBS, lysed, and stored temporarily at −20 °C ahead of extraction. The Copan 4N6FLOQ Buccal Swabs were cut and lysed sequentially in the same aliquot of lysis buffer prior to temporary storage at −20 °C ahead of extraction. Whole blood samples were simply held transiently at −20 °C until DNA extraction. DNA was extracted from whole blood, cervical, and buccal tissue lysates on a Hamilton Star liquid handling platform using the Nucleo-Mag Blood 200 µl kit (Macherey Nagel, cat #744501.4) with prior modifications for optimal lysis of cervical cell pellets and paired buccal swabs. For breast tissues, DNA was extracted from up to 40 mg of tissue using the Lipid Tissue kit from Macherey Nagel (cat # 740471.50), and the manufacturer’s instructions were followed. DNA concentration and quality absorbance ratios were measured using Nanodrop-8000, Thermoscientific Inc. Extracted DNA was stored at −80 °C until further analysis.

### DNA methylation analysis

Cervical, buccal, and breast tissue DNA were normalised to 25 ng/µL and 500 ng total DNA was bisulfite modified using the EZ-96 DNA Methylation-Lightning kit (Zymo Research Corp, cat #D5047) on the Hamilton Star Liquid handling platform. 8 µL of modified DNA was subjected to methylation analysis on the Illumina InfiniumMethylation EPIC BeadChip (Illumina, CA, USA) at UCL Genomics according to the manufacturer’s standard protocol.

All methylation microarray data were processed through the same standardised pipeline running in R version 4.0.2. Raw data were loaded using the R package minfi, version 1.36.0. Any samples with median methylated and unmethylated intensities < 9.5 were removed. Any probes with a detection p-value > 0.01 were regarded as having failed. Any samples with >10% failed probes, and any probes with >10% failure rate were removed from the dataset. Beta values from failed probes (approximately 0.001% of the dataset) were imputed using the impute.knn function as part of the impute R package, version 1.62.0. Non-CpG probes (2932), SNP-related probes as identified by Zhou et al.^43^ (82,108), and chrY probes were removed from the dataset. An additional 6102 previously identified probes that followed a trimodal methylation pattern characteristic of an underlying SNP were removed.

Background intensity correction and dye bias correction were performed using the minfi single sample preprocessNoob function. Probe bias correction was performed using the beta mixture quantile normalisation (BMIQ) algorithm of the ChAMP package, version 2.18.3.

The fraction of immune cell contamination, and the relative proportions of different immune cell subtypes in each sample, were estimated using the EpiDISH algorithm utilising the epithelial, fibroblast and immune cell reference dataset, version 2.6.1. The top 1000 most variable probes (ranked by standard deviation) were used in a principal component analysis. Statistical tests were performed in order to identify any anomalous associations between plate, sentrix position, date of array processing, date of DNA creation, study centre, immune contamination fraction, age, type (case versus control), and the top ten principal components. Finally, two-thirds of the discovery dataset was randomly selected for use as the training dataset and the remaining third was allocated to the internal validation dataset.

### Statistical analyses for classifier development

mQTL data was obtained from Supplementary Table 5 of the supplementary information of a recent publication by Ho et al.^33^. The 822 probes at loci associated with breast cancer risk were extracted, of which 704 unique CpGs were present in the EPIC array datasets after QC and filtering. The Discovery set was randomly split 70–30% into a training and internal validation set. The R package glmnet, version 4.1.3, was used to train L1 or L2 regularised classifiers (lasso or ridge regression) based on these 704 mQTL-associated CpGs in the training set. Ten-fold cross-validation was used in the training set via the cv.glmnet function in order to determine the optimal value of the regularisation parameter lambda. The AUC in the internal validation set, computed using the pROC package, version 1.18.0, was used as a metric of classifier performance. The final classifier was selected based on the highest AUC obtained in the internal validation dataset. The training and internal validation datasets were then combined, and the classifier was refitted using the entire discovery dataset using lasso regularisation. This finalised classifier was then applied to the external validation dataset and the corresponding AUC was computed.

Denoting the 104 selected mQTL CpGs as ${\beta }_{1},\ldots ,{\beta }_{n}$ and the regression coefficients from the trained classifier as ${w}_{1},\ldots ,{w}_{n}$, then WID™-qtBC index is (Eq. 1)

1 $$\mathop{\sum }\limits_{i=1}^{n}{(w}_{i}{\beta }_{i}-\mu )/\sigma$$

where *μ* and *σ* are defined as the mean and standard deviation of the quantity ${\mathop{\sum }\nolimits_{i=1}^{n}}{w}_{i}{\beta }_{i}$ in the Discovery dataset (that is, the index is scaled to have zero mean and unit standard deviation in the Discovery dataset). The included CpGs and coefficients are displayed in Supplementary Table 4.

### SNP genotyping, QC, and computation of the polygenic risk score

Genotyping of 318 breast cancer cases and 850 controls from the discovery set and 113 breast cancer cases and 225 controls in the validation set was conducted using the Illumina 650k Infinium Global Screening Array. Whole blood DNA was normalised to 75 ng/µL and Global Screening Array (GSA). Whole blood DNA was normalised to 75 ng/µL and a total of 300 ng applied to the Infinium Global Screening Array – 24 V2 (Illumina, CA, USA) at UCL Genomics according to the manufacturer’s standard protocol. Genotype calling was performed using GenomeStudio, with genetic variants found to be clustering poorly being removed from further analyses. One control subject from the Discovery set failed to genotype. For duplicate genetic variant pairs, the variant within each pair with the lowest calling and clustering score was excluded. Autosomal SNPs were used in subsequent QC and PRS_313_ analyses (except for checks for sex mismatches, where the X chromosome was used to infer sex).

General subject and single nucleotide polymorphism (SNP) quality control (QC) was performed using PLINK version 1.9^44^. In the Discovery set, three breast cancer cases and eight controls with a call rate less than 95% were excluded, and one breast cancer and three controls were further removed due to genetically inferred sex not being female. No subjects failed QC in the Validation set. Genetic variants with missing genotype rate greater than 5%, minor allele frequency (MAF) less than 1%, or a significant departure from Hardy-Weinberg equilibrium (*p* < 5 × 10^−6^) were excluded. KING^45^, a relatedness inference algorithm, was next used to identify duplicate/monozygotic twin or first-degree relative pairs. In the Discovery set, one control subject pair was identified as being a duplicate/monozygotic twin pair, and nine control pairs were inferred to be first-degree relatives. In the Validation set, one control pair was inferred to be first-degree relatives. The subject in each related pair with the lower call rate was excluded. In the Discovery set, 314 breast cancer subjects and 816 controls, and 479,105 variants were retained after SNP QC, of which 809 and 312 overlapped with samples that passed QC for methylation. In the Validation set, 113 breast cancer cases and 224 controls, and 501,209 variants passed QC, of which 210 and 111 overlapped with samples that passed QC for methylation.

Non-European subjects were identified by plotting the top two principal components, generated using GTCA version 1.93.2, for the Discovery or Validation set, respectively, and 270 HapMap phase II release 23 samples (CEU, YRI, JPT, and CHB individuals), downloaded in PLINK-formatted binary files from http://zzz.bwh.harvard.edu/plink/res.shtml. Subjects not found to cluster around HapMap European samples were excluded from further analysis. After excluding non-European subjects, 312 breast cancer cases and 809 controls were retained in the Discovery set, while 111 breast cancer cases and 210 controls were retained in the Validation set.

Using the Michigan Imputation Server^46^ and the 1000 Genomes Phase 3 reference panel, the SNP discovery dataset went through further QC before being phased (Eagle2) and imputed. Variants were strand, allele, genetic position, or allele frequencies were not concordant with the 1000 Genomes Phase 3 reference panel were removed before phasing and imputation using Strand Tools. After imputation, exclusion of variants with imputation R^2^ < 0.5 and removal of variants with 3 or more alleles, both the Discovery and Validation set contained 303 of the 313 SNPs used by Mavaddat et al.^4^ to develop a 313 SNP breast cancer polygenic risk score (PRS_313_) were imputed. Of note, only 293 SNPs of the 303 SNPs in each set were overlapping between the Discovery and Validation set. We constructed the breast cancer PRS_313_ for each subject as follows (Eq. 2):

2 $${{PRS}}_{j}=\mathop{\sum }\limits_{i=1}^{303}{\hat{{\beta }}_{i}}{x}_{{ij}}$$

where, $\hat{{\beta }_{i}}$ is the log odds ratio for the *i*-th SNP taken from publicly available Oncoarray summary association results (combined Oncoarray, iCOGs and BCAC overall breast cancer beta values) in the publication by Mavaddat et al. and *x*_*ij*_ is the number of copies of the effect allele present in each discovery cohort subject. Scores were generated using PLINK version 1.9.

### High and low risk stratification

Stratification into high and low PRS_313_ or WID™-qtBC values was conducted by computing the median PRS_313_ or WID™-qtBC across all samples in a given set. High PRS_313_ or WID™-qtBC scores were defined as above the median, while low PRS_313_ or WID™-qtBC scores were equal to or below the median. For risk stratification using both PRS_313_ and WID™-qtBC, the groupings (high/low) as described above were combined.

### Reporting summary

Further information on research design is available in the Nature Research Reporting Summary linked to this article.

### Supplementary information

Supplementary Information

Supplementary Table 3.

Reporting Summary

## Data Availability

The DNAme data generated in this study have been deposited in the European Genome-phenome Archive (EGA) database under accession code EGAS00001005055. Additional data is available on Gene Expression Omnibus under the accession number GSE133985.

## Code availability

Code to reproduce analysis in this study is found under https://github.com/chiaraherzog/WID_qtBC/.
