# Supplementary material for: DNA methylation at quantitative trait loci (mQTLs) varies with cell type and nonheritable factors and may improve breast cancer risk assessment
Source: NPJ Precis Oncol. 2023 Sep 27;7:99. doi: 10.1038/s41698-023-00452-2 (PMC10533818; doi:10.1038/s41698-023-00452-2)
Supplement: Supplementary file 1 — Supplementary Information [file 41698_2023_452_MOESM1_ESM.pdf]

DNA methylation at quantitative trait loci (mQTLs) varies with cell type and non-heritable factors and may improve breast cancer risk assessment

Supplementary Information

Chiara Herzog, Allison Jones, Iona Evans, Michal Zikan, David Cibula, Nadia Harbeck, Nicoletta Colombo, Angelique Flöter Rådestad, Kristina Gemzell-Danielsson, Nora Pashayan and Martin Widschwendter

Contents

Supplementary Information **2**

Supplementary Table 1. Study populations and characteristics. 2

Supplementary Table 2. Clinical characteristics of cancer cases in the discovery and validation sets. 3

Supplementary Table 3. Information on mQTL CpGs. 4

Supplementary Table 4. Stratification by the PRS_{313} in the discovery and validation sets. 5

Supplementary Table 5. Stratification by WID™-qtBC in the validation set. 6

Supplementary Figure 1. Overview of discovery and validation sets for index development and SNP availability. 7

Supplementary Figure 2. Association of genetic and non-genetic factors on WID™-qtBC. 8

Supplementary Information

Supplementary Table 1. Study populations and characteristics.

	n	Accession ID	Age	Tissue	Premenopausal (%)	PRS_{313} available (n)
**breast variability set**						
Normal-adjacent	14 (28%)	EGAD00010002074	44 (38.75-46.75)	breast tissue	14 (100%)	0
Normal	14 (28%)	EGAD00010002075	27 (24-39.5)	breast tissue	14 (100%)	0
BRCA1	10 (20%)	EGAD00010002075	35 (33.25-38)	breast tissue	10 (100%)	0
BRCA2	4 (8%)	EGAD00010002075	37 (34.5-40.25)	breast tissue	4 (100%)	0
Normal-adjacent	8 (16%)	GSE133985	49.5 (45-54.33)	breast tissue	Unknown	0
**matched surrogate samples**						
Healthy volunteer	222 (100%)	EGAS00001005626	40.55 (31.68-48.7)	cervical, buccal, and blood sample	164 (73.87%)	0
**discovery set**						
Control	869 (72.54%)	EGAS00001005055	47.33 (38.61-57.36)	cervical sample	509 (58.57%)	809
Breast cancer	329 (27.46%)	EGAS00001005055	52.43 (45.63-62.67)	cervical sample	159 (48.33%)	312
**validation set**						
Control	225 (66.57%)	EGAS00001005055	54.19 (45.33-61.23)	cervical sample	79 (35.11%)	210
Breast cancer	113 (33.43%)	EGAS00001005055	51.14 (45.67-63.51)	cervical sample	57 (50.44%)	111
**breast validation set**						
Normal-adjacent	14 (28%)	EGAD00010002074	44 (38.75-46.75)	breast tissue	14 (100%)	0
Normal	14 (28%)	EGAD00010002075	27 (24-39.5)	breast tissue	14 (100%)	0
Normal-adjacent	8 (16%)	GSE133985	49.5 (45-54.33)	breast tissue	8 (100%)	0
**breast treatment set**						
Mifepristone	18 (45%)	EGAD00010002073	25 (24-37)	breast biopsy	18 (100%)	0
Vitamin	22 (55%)	EGAD00010002073	25 (23.25-29.5)	breast biopsy	22 (100%)	0

Supplementary Table 2. Clinical characteristics of cancer cases in the discovery and validation sets.

Characteristic	discovery set		validation set	
	SNP data available n = 312	SNP data not available n = 17	SNP data available n = 111	SNP data not available n = 2
**ER status**				
ER+	267 (86%)	15 (88%)	92 (83%)	2 (100%)
ER-	44 (14%)	2 (12%)	17 (15%)	0 (0%)
Unknown	1 (0.3%)	0 (0%)	2 (1.8%)	0 (0%)
**PR status**				
PR+	233 (75%)	14 (82%)	80 (72%)	2 (100%)
PR-	76 (24%)	3 (18%)	27 (24%)	0 (0%)
Unknown	3 (1.0%)	0 (0%)	4 (3.6%)	0 (0%)
**HER2 status**				
HER2+	103 (96%)	4 (100%)	47 (98%)	1 (100%)
HER-	0 (0%)	0 (0%)	0 (0%)	0 (0%)
Unknown	4 (3.7%)	0 (0%)	1 (2.1%)	0 (0%)
Unknown	205	13	63	1
**Nodal stage**				
N0	144 (46%)	9 (53%)	49 (44%)	0 (0%)
N1	109 (35%)	7 (41%)	41 (37%)	2 (100%)
N2	30 (9.6%)	0 (0%)	14 (13%)	0 (0%)
N3	20 (6.4%)	1 (5.9%)	5 (4.5%)	0 (0%)
Unknown	9 (2.9%)	0 (0%)	2 (1.8%)	0 (0%)
**Tumour stage**				
T1	133 (43%)	9 (53%)	50 (45%)	1 (50%)
T2	155 (50%)	8 (47%)	48 (43%)	1 (50%)
T3	20 (6.4%)	0 (0%)	12 (11%)	0 (0%)
T4	2 (0.6%)	0 (0%)	1 (0.9%)	0 (0%)
Unknown	2 (0.6%)	0 (0%)	0 (0%)	0 (0%)
**Tumour grade**				
Grade I	20 (6.4%)	1 (5.9%)	8 (7.2%)	0 (0%)
Grade II	128 (41%)	7 (41%)	50 (45%)	1 (50%)
Grade III	161 (52%)	9 (53%)	53 (48%)	1 (50%)
Unknown	3 (1.0%)	0 (0%)	0 (0%)	0 (0%)

Supplementary Table 3. Information on mQTL CpGs.

See Supplementary Table 3 provided as .xlsx.

Supplementary Table 4. Stratification by the PRS_{313} in the discovery and validation sets.

P values and odds ratios were estimated using median-unbiased estimation. Statistically significant values are shown in bold.

Risk group	Cases (n)	Controls (n)	Odds ratio	95% CI	p value
**Discovery set**					
low PRS_{313}	105	456	1 (Reference)	-	-
high PRS_{313}	207	353	**2.5**	**1.9-3.4**	**p=8.13e-12**
**Validation set**					
low PRS_{313}	38	123	1 (Reference)	-	-
high PRS_{313}	73	87	**2.7**	**1.7-4.4**	**p=3.80e-05**

Low and **high** grouping was defined on median values of the PRS_{313} in each set. **High** scores were above the median, while **low** scores were defined as equal to or below the median.

Supplementary Table 5. Stratification by WID™-qtBC in the validation set.

P values and odds ratios were estimated using median-unbiased estimation. Statistically significant values are shown in bold.

Risk group	Cases (n)	Controls (n)	Odds ratio	95% CI	p value
**Validation set**					
low WID™-qtBC	32	129	1 (Reference)	-	-
high WID™-qtBC	79	81	**3.9**	**2.4-6.5**	**p=2.67e-08**

Low and **high** grouping was defined on median values of the WID™-qtBC in each set. **High** scores were above the median, while **low** scores were defined as equal to or below the median.

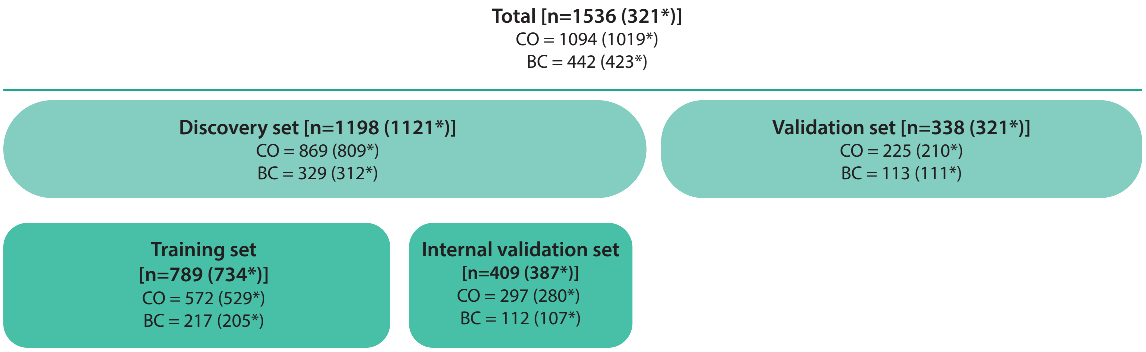

Supplementary Figure 1. Overview of discovery and validation sets for index development and SNP availability.

Abbreviations: CO, number of controls. BC, number of breast cancer cases. * indicates n for which SNP data is available.

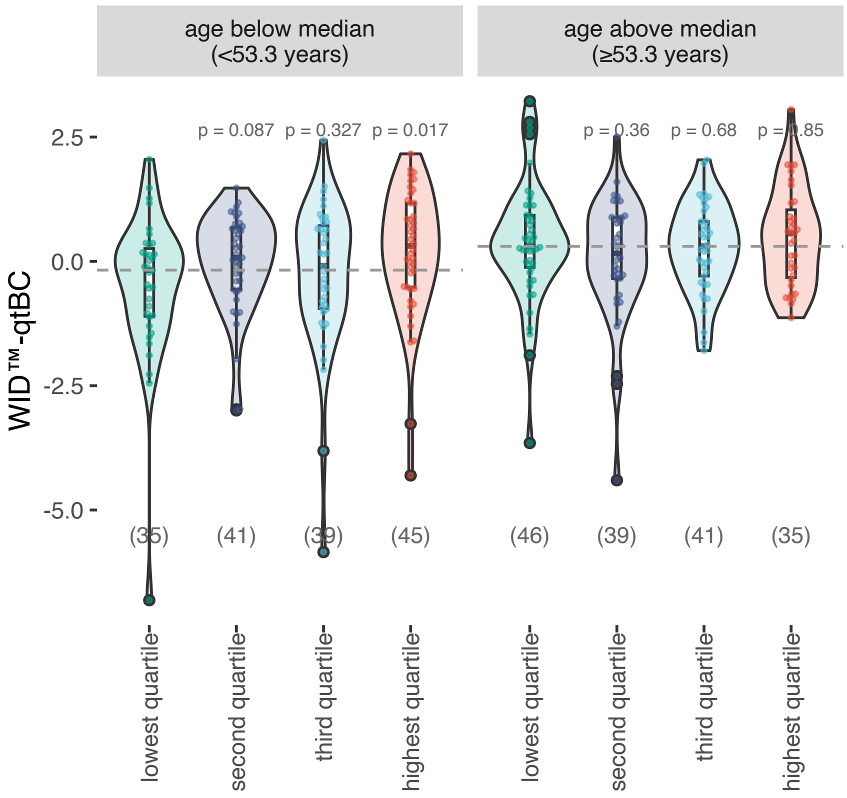

Supplementary Figure 2. Association of genetic and non-genetic factors on WID™-qtBC.

Stratification by age and PRS_{313} levels in the external validation set reveals that a higher PRS_{313} drives a higher mQTL-based risk particularly in younger age (p = 0.017 comparison to lowest quartile in younger age group, Wilcoxon test). Boxplot boxes indicate median (centre line), interquartile range (bounds of box), and 95% confidence interval (whiskers).
