# Supplementary material for: DNA methylation at quantitative trait loci (mQTLs) varies with cell type and nonheritable factors and may improve breast cancer risk assessment
Source: NPJ Precis Oncol. 2023 Sep 27;7:99. doi: 10.1038/s41698-023-00452-2 (PMC10533818; doi:10.1038/s41698-023-00452-2)
Supplement: Supplementary file 3 — Reporting Summary [file 41698_2023_452_MOESM3_ESM.pdf]

nature portfolio

Corresponding author(s): Martin Widschwendter

Last updated by author(s): 2023/09/06

Reporting Summary

Nature Portfolio wishes to improve the reproducibility of the work that we publish. This form provides structure for consistency and transparency in reporting. For further information on Nature Portfolio policies, see our Editorial Policies and the Editorial Policy Checklist.

Statistics

For all statistical analyses, confirm that the following items are present in the figure legend, table legend, main text, or Methods section.

n/a	Confirmed	
☐	☒	The exact sample size (*n*) for each experimental group/condition, given as a discrete number and unit of measurement
☐	☒	A statement on whether measurements were taken from distinct samples or whether the same sample was measured repeatedly
☐	☒	The statistical test(s) used AND whether they are one- or two-sided<br>*Only common tests should be described solely by name; describe more complex techniques in the Methods section.*
☐	☒	A description of all covariates tested
☒	☐	A description of any assumptions or corrections, such as tests of normality and adjustment for multiple comparisons
☒	☐	A full description of the statistical parameters including central tendency (e.g. means) or other basic estimates (e.g. regression coefficient) AND variation (e.g. standard deviation) or associated estimates of uncertainty (e.g. confidence intervals)
☐	☒	For null hypothesis testing, the test statistic (e.g. *F*, *t*, *r*) with confidence intervals, effect sizes, degrees of freedom and *P* value noted<br>*Give P values as exact values whenever suitable.*
☒	☐	For Bayesian analysis, information on the choice of priors and Markov chain Monte Carlo settings
☒	☐	For hierarchical and complex designs, identification of the appropriate level for tests and full reporting of outcomes
☐	☒	Estimates of effect sizes (e.g. Cohen's *d*, Pearson's *r*), indicating how they were calculated

Our web collection on statistics for biologists contains articles on many of the points above.

Software and code

Policy information about availability of computer code

Data collection	Epidemiological data from the FORECEE Study were obtained via Qualtrics questionnaire. Additional data on studies were obtained by clinical cooperation partners or from publicly available datasets (e.g., GEO). Methylation data were obtained via Illumina iScan and processed further using the R programming environment.
Data analysis	All data were analysed in the R programming environment (version 4.2.2 (2022-10-31)). All code is provided under https:// github.com/ chiaraherzog/WID_qtBC/.

For manuscripts utilizing custom algorithms or software that are central to the research but not yet described in published literature, software must be made available to editors and reviewers. We strongly encourage code deposition in a community repository (e.g. GitHub). See the Nature Portfolio guidelines for submitting code & software for further information.

Data

Policy information about availability of data

All manuscripts must include a data availability statement. This statement should provide the following information, where applicable:

- Accession codes, unique identifiers, or web links for publicly available datasets
- A description of any restrictions on data availability
- For clinical datasets or third party data, please ensure that the statement adheres to our policy

The DNAme data generated in this study have been deposited in the European Genome-phenome Archive (EGA) database under accession code EGAS00001005055. Additional data is available on Gene Expression Omnibus under the accession number GSE133985.

Research involving human participants, their data, or biological material

Policy information about studies with human participants or human data. See also policy information about sex, gender (identity/presentation), and sexual orientation and race, ethnicity and racism.

Reporting on sex and gender	Our analyses focused on breast cancer and hence warrant a sex-specific analysis (only women were included in this study).
Reporting on race, ethnicity, or other socially relevant groupings	Data were obtained from previously published studies or trials and data collections centered around Europe (various study centers around Europe). Details on these studies are provided in the methods section, and referenced appropriately.
Population characteristics	Detailed population characteristics, including age and menopausal status are described, where available, in the Extended/ Supplementary data of the manuscript, and previous publications.
Recruitment	Recruitment is outlined in initial study publications.
Ethics oversight	For the FORECEE programme, ethical approval was provided from the UK Health Research Authority (REC 14/LO/1633) and other contributing centres. For the breast tissue samples ethical approval was obtained from the NRES Committee East of England (reference number 15/EE/0192). The Mifepristone trial was approved by the regional ethical review board at Karolinska Institutet permit 2009/144-31/4 and the regional ethical review board at Karolinska Institutet permit 2012/729 31/1.

Note that full information on the approval of the study protocol must also be provided in the manuscript.

Field-specific reporting

Please select the one below that is the best fit for your research. If you are not sure, read the appropriate sections before making your selection.

☒ Life sciences ☐ Behavioural & social sciences ☐ Ecological, evolutionary & environmental sciences

For a reference copy of the document with all sections, see nature.com/documents/nr-reporting-summary-flat.pdf

Life sciences study design

All studies must disclose on these points even when the disclosure is negative.

Sample size	No sample size calculation was conducted for this specific study, although sample size calculations were conducted for the underlying studies, described elsewhere (referenced in the manuscript text).<br>We used data available from previous sample collections, including one of the largest IlluminaMethylationEPIC array methylation datasets to date on cervical samples, as well as samples available on matched tissues.
Data exclusions	No data were excluded from this study.
Replication	No replication studies were possible within the current study, given limited sample access (e.g., to samples from the mifepristone study).
Randomization	n/a
Blinding	n/a

Reporting for specific materials, systems and methods

We require information from authors about some types of materials, experimental systems and methods used in many studies. Here, indicate whether each material, system or method listed is relevant to your study. If you are not sure if a list item applies to your research, read the appropriate section before selecting a response.

Materials & experimental systems

n/a	Involved in the study
☒	☐ Antibodies
☒	☐ Eukaryotic cell lines
☒	☐ Palaeontology and archaeology
☒	☐ Animals and other organisms
☐	☒ Clinical data
☒	☐ Dual use research of concern
☒	☐ Plants

Methods

n/a	Involved in the study
☒	☐ ChIP-seq
☒	☐ Flow cytometry
☒	☐ MRI-based neuroimaging

Clinical data

Policy information about clinical studies

All manuscripts should comply with the ICMJE guidelines for publication of clinical research and a completed CONSORT checklist must be included with all submissions.

Clinical trial registration	n/a (previously published: "Mifepristone treatment prior to insertion of a levonorgestrel releasing intrauterine system for improved bleeding control – a randomized controlled trial" (EudraCT number: 2009-009014-40))
Study protocol	n/a
Data collection	n/a, previously published
Outcomes	n/a
